# Therapeutic Advantages of Isoflavone Glycoside and Aglycone Forms of Sophoricoside in the Amelioration of Postmenopausal Symptoms: Bone Health, Metabolic Regulation, and Systemic Inflammation

**DOI:** 10.3390/molecules30102218

**Published:** 2025-05-20

**Authors:** Jeong-Won Ahn, Hyun-Soo Kim, Kongara Damodar, Hee-Hyun Shin, Kyung-Mi Kim, Jung-Youl Park, Yeong-Min Yoo, Jae-Chul Jung, Seong-Soo Joo

**Affiliations:** 1 Department of Marine Bioscience, College of Life Science, Gangneung-Wonju National University, Gangneung 25457, Gangwon, Republic of Korea; 0000@gwnu.ac.kr (J.-W.A.); gustn4609@gwnu.ac.kr (H.-S.K.); 2 Huscion MAJIC R&D Center, 331 Pangyo-ro, Seongnam 13488, Gyeonggi, Republic of Korea; kongaradamu@gwnu.ac.kr; 3 Life Science Research Institute, NOVAREX Co., Ltd., Cheongju 28220, Chungbuk, Republic of Korea; hhshin@novarex.co.kr (H.-H.S.); kkm3507@novarex.co.kr (K.-M.K.); jcjung@novarex.co.kr (J.-C.J.); 4 Glocal University Project Group, Andong National University, 1375 Gyeongdong-ro, Andong 36729, Gyeongbuk, Republic of Korea; jypark09@anu.ac.kr; 5 Environmental Research Institute, Kangwon National University, Chuncheon-si 24341, Gangwon, Republic of Korea; yyeongm@hanmail.net

**Keywords:** postmenopausal syndrome, phytoestrogens, *Styphnolobium japonicum*, sophoricoside, genistein, biotransformation

## Abstract

This study investigates the therapeutic potential of sophoricoside and its aglycone metabolite, genistein, derived from *Styphnolobium japonicum* L. fruit, as natural alternatives to hormone replacement therapy for postmenopausal symptom management. Using *Lactobacillus plantarum* to model intestinal biotransformation, we compared glycoside-rich (Rex) and aglycone-rich (Rex-AG) extracts in ovariectomized rats. Both treatments significantly reduced weight gain and alleviated vaginal dryness, with Rex demonstrating superior thermoregulatory stabilization. Histological and molecular analyses revealed preserved trabecular bone integrity through the downregulation of RANKL and upregulation of TGF-β. Both extracts exhibited potent anti-inflammatory effects in adipose tissue, suppressing IL-6 and TNF-α, while regulating adipogenesis markers (FABP4, KLF, leptin, PPARγ) more effectively than 17β-estradiol. Serum genistein concentrations confirmed its efficient biotransformation and systemic bioavailability. Importantly, the treatments showed favorable safety profiles with no adverse effects on organ weight. These findings establish *S. japonicum* L. fruit-derived phytoestrogens as promising candidates for the comprehensive management of postmenopausal symptoms, offering an efficacious and safer alternative to conventional hormone therapy.

## 1. Introduction

Menopause signifies the permanent cessation of menstruation, which is accompanied by a wide range of physical and emotional symptoms driven by hormonal changes. Common symptoms include hot flashes, night sweats, mood swings, and an increased risk of osteoporosis and metabolic disorders [1]. While hormone replacement therapy (HRT) remains a primary intervention for these symptoms, its long-term use has raised concerns about severe side effects, such as an increased risk of breast cancer, stroke, and cardiovascular disease [2]. Consequently, the need for safer, more natural alternatives has driven growing interest in non-hormonal therapies [3,4]. Phytoestrogens, plant-derived compounds that mimic estrogen activity, have gained attention as viable alternatives to conventional HRT. Among these, isoflavones are particularly notable for their potential to reduce the frequency and severity of hot flashes, improve bone health, and mitigate other menopause-related risks [5].

Sophoricoside, an isoflavone glycoside found abundantly in *Styphnolobium japonicum* L. fruit, has demonstrated promising phytoestrogenic properties. Upon ingestion, sophoricoside is metabolized by gut microbiota into genistein, an aglycone with significantly enhanced bioactivity and bioavailability [6,7]. This biotransformation, facilitated by intestinal microbes producing β-glucosidase, is crucial for its therapeutic efficacy [8].

Traditionally, *S. japonicum* L. has been documented in East Asian pharmacopoeias as “Huai Jiao” or “Huai Shi,” described as having cold properties with bitter and astringent tastes. These characteristics have been considered ideal for cooling heat and calming blood, which correlate with the anti-inflammatory, anti-hypertensive, and antioxidative properties of this plant, which have been confirmed through modern scientific studies [9,10]. Contemporary studies have demonstrated the potential of *S. japonicum* L. fruit extracts in alleviating menopausal symptoms, particularly by mitigating osteoporosis and metabolic dysfunctions [11,12,13]. However, the comparative biological activities of sophoricoside and its aglycone metabolite, genistein, in addressing postmenopausal symptoms remain inadequately explored. Understanding their distinct roles and mechanisms could provide a foundation for developing novel therapies.

This study aims to address this gap by evaluating the efficacy of *S. japonicum* L. fruit extracts (Rex) and their genistein-enriched aglycone form (Rex-AG) in managing postmenopausal symptoms. Using an ovariectomized (OVX) rat model, a widely accepted experimental system mimicking estrogen-deficient conditions, we investigated the effects of these treatments on body weight regulation, thermoregulation, vaginal health, bone density, and inflammatory markers. By elucidating their mechanisms of action, this study provides new insights into the potential use of *S. japonicum* L. fruit as a functional food or natural alternative to conventional HRT.

## 2. Results and Discussion

### 2.1. Screening and Selection of Probiotic Strains for Biotransformation

The selection of an optimal probiotic strain is critical for achieving efficient biotransformation of bioactive compounds and for supporting gut health. In this study, we focused on the characteristics of *L. plantarum*, a strain widely recognized for its natural presence in the human gastrointestinal tract and its contributions to host physiological well-being [14,15,16]. Its robust growth and metabolic activity in nutrient-rich environments, such as those enriched with PAE, have demonstrated its ability to utilize sugar as a primary energy source. This capability not only highlights its adaptability in complex substrates but also suggests its potential role in promoting gut microbiota balance, immune modulation, and nutrient absorption [17,18].

**Figure 1:**
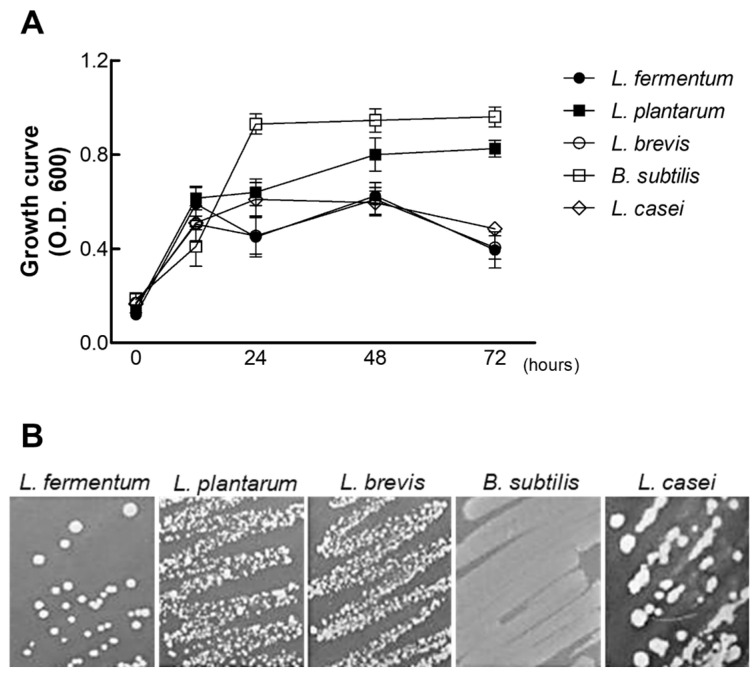
Comparative growth analysis of five biocompatible bacterial strains in *Panax ginseng* extract (PAE). (**A**) Growth curves of five bacterial strains (*L. fermentum, L. plantarum, L. brevis, L. casei,* and *B. subtilis*) over 72 h in PAE. (**B**) Visual and quantitative assessment of bacterial growth after 72 h, conducted by streaking onto selective solid media. Data represent the mean ± standard deviation from three independent experiments.

A comparative analysis of multiple probiotic strains, including *L. fermentum*, *L. brevis*, *L. casei*, and *B. subtilis*, revealed that *L. plantarum* exhibited superior biotransformation efficiency for converting ginsenosides in PAE into ginsenoside Rg3. The ginsenosides Rb1, Rc, and Rd are particularly susceptible to enzymatic deglycosylation, a process facilitated by the β-glucosidase activity inherent in probiotics [19,20]. Over 72 h of fermentation at 35 °C, both *L. plantarum* and *B. subtilis* demonstrated stable growth (Figure 1), but *L. plantarum* achieved significantly higher Rg3 yields after 48 h, as confirmed by HPLC analysis (Table 1 and Supplementary Figure S2). These findings underscore its robust enzymatic activity and biotransformation capabilities, which make it an ideal candidate in further applications for converting sophoricoside into its aglycone form, genistein. This investigation establishes *L. plantarum* not only as a highly effective strain for biotransformation but also as a promising probiotic for enhancing functional food products targeting postmenopausal health. Its ability to facilitate the conversion of glycosylated bioactive compounds into their more bioavailable forms aligns with its established benefits for gut health and systemic physiological functions. These attributes provide a strong foundation for its application in the development of biotransformed phytoestrogens, such as genistein, which hold therapeutic potential for addressing menopausal symptoms naturally.

### 2.2. Fermentation-Based Biotransformation of Sophoricoside to Genistein

**Figure 2:**
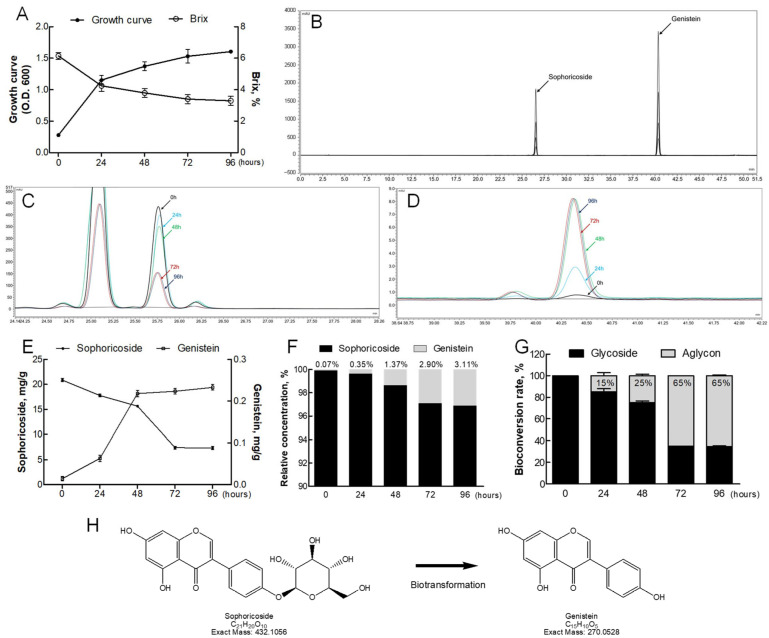
Biotransformation efficiency of *L. plantarum* in Rex extract. (**A**) Growth curve of *L. plantarum* over time, with a decrease in Brix values reflecting sugar consumption. (**B**) HPLC chromatograms of sophoricoside and genistein standards as reference. (**C**,**D**) HPLC peak changes for sophoricoside and genistein during fermentation. (**E**) Quantification of the sophoricoside-to-genistein conversion. (**F**) Biotransformation efficiency (%) of sophoricoside to genistein over 96 h of fermentation. Values represent means from three independent experiments (n = 3): 0 h (0.07 ± 0.01%), 24 h (0.35 ± 0.05%), 48 h (1.37 ± 0.18%), 72 h (2.90 ± 0.32%), and 96 h (3.11 ± 0.28%). (**G**) Relative transformation rate for glycoside (sophoricoside) to aglycone (genistein). (**H**) Schematic of deglycosylation by *L. plantarum*. Data are presented as the mean ± standard (n = 3). Rex, *S. japonicum* L. fruit extract.

The biotransformation of sophoricoside to genistein during the fermentation of Rex (glycosylated form) and Rex-AG (aglycone-enriched form) by *L. plantarum* was systematically evaluated to elucidate the metabolic potential of this strain. Growth-curve analysis demonstrated stable proliferation of *L. plantarum*, with a significant reduction in sugar Brix values, which reached their lowest levels after 72 h of fermentation (Figure 2A). This decrease in Brix levels reflects efficient sugar utilization, further confirming the metabolic activity of *L. plantarum* during the fermentation process.

HPLC analysis was employed to monitor the conversion of sophoricoside to genistein. The retention times for sophoricoside and genistein were approximately 25.6 and 40.3 min, respectively (Figure 2B). Quantitative analysis revealed a marked decrease in sophoricoside levels and a corresponding increase in genistein levels over the 72 h fermentation period (Figure 2C–E). The transformation efficiency was quantified as approximately 3.11%, with the ratio of glycoside to aglycone shifting from being predominantly glycoside to a 35:65 glycoside–aglycone balance after fermentation (Figure 2F,G). These findings indicate that *L. plantarum* effectively facilitates the deglycosylation of sophoricoside into its bioactive aglycone form, genistein. Additionally, detailed insights into the fermentation-based deglycosylation process were obtained (Figure 2H), providing robust evidence for the role of *L. plantarum* in catalyzing this conversion. The efficient transformation highlights the enzymatic activity of β-glucosidase, a key enzyme responsible for breaking down glycosidic bonds, thereby enhancing the bioavailability and therapeutic potential of sophoricoside [21].

This comprehensive evaluation underscores the innovative application of probiotics in biotransforming bioactive compounds into more bioavailable forms. The ability of *L. plantarum* to facilitate the conversion of sophoricoside to genistein not only strengthens its role in functional food development but also positions Rex and Rex-AG as promising candidates for managing postmenopausal symptoms. By improving the bioactivity of these phytoestrogens through fermentation, this study lays the groundwork for developing next-generation functional foods and natural therapeutic agents for health management.

### 2.3. Effects of Treatment on Body Weight and Thermoregulatory Symptoms in OVX Rats

**Figure 3:**
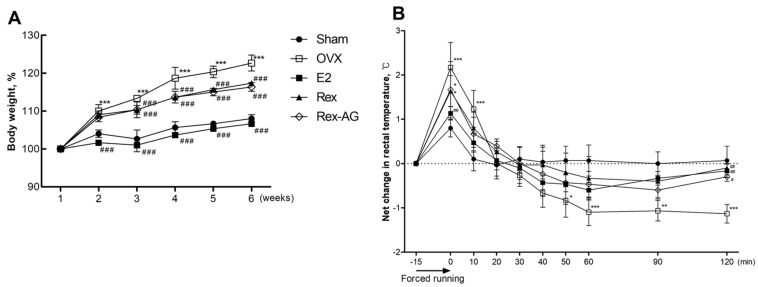
Assessment of menopausal symptoms in ovariectomized (OVX) rats. Rats were administered 150 mg/kg/day of Rex or Rex-AG for six weeks, with 17β-estradiol (E2) as a positive control. (**A**) Body weight changes over the six-week treatment period. (**B**) Rectal temperature changes measured after forced exercise at the end of the six-week treatment, indicating thermoregulatory effects of the treatments. Data are presented as the mean ± standard deviation (n = 7). Statistical significance is indicated as * *p* < 0.05, ** *p* < 0.01 and *** *p* < 0.001 compared with the sham group or # *p* < 0.05, ## *p* < 0.01 and ### *p* < 0.001 compared with the OVX group. Rex, *S. japonicum* L. fruit extract (glycoside form); Rex-AG, Rex aglycone form.

To evaluate key postmenopausal features in an OVX SD rat model, this study monitored changes in body weight and vasomotor symptoms over a six-week period. In this study, 17β-estradiol (E2) was selected as a positive control for several critical reasons. As the primary and most potent endogenous estrogen in mammals, 17β-estradiol represents the gold standard for evaluating estrogenic effects in experimental models of menopause. Its established efficacy in hormone replacement therapy for postmenopausal women provides a well-characterized reference point against which to compare our phytoestrogen-based interventions. The estrogen-supplemented positive-control group exhibited minimal weight gain, emphasizing the critical role of estrogen in maintaining metabolic balance and preventing excessive weight gain after an ovariectomy. Similarly, both the Rex and Rex-AG treatment groups showed significantly lower weight increases compared with the untreated OVX control group (Figure 3A). These results suggest that Rex and Rex-AG effectively mimic the weight-regulatory effects of estrogen, consistent with previous findings in post-ovariectomy rodent models [22,23]. The reduction in weight gain highlights the potential metabolic benefits of these treatments in postmenopausal states. In addition to weight regulation, thermoregulatory stability was assessed as an indicator of vasomotor symptoms, such as hot flashes, using rectal temperature measurements. OVX rats displayed pronounced rectal temperature fluctuations, with elevated temperatures persisting for more than two hours, indicative of thermoregulatory dysfunction associated with estrogen deficiency. Notably, the Rex-treated group exhibited smaller temperature fluctuations compared with both the Rex-AG and OVX control groups (Figure 3B). This response closely mirrored the pattern observed in the estrogen-supplemented positive control group, suggesting that Rex treatment is particularly effective in mitigating vasomotor symptoms. The distinct effects of Rex and Rex-AG on thermoregulation may reflect differences in their metabolic profiles and bioactive compound composition. Rex, as a glycosylated extract, may have a more sustained release of active metabolites compared with Rex-AG, which is enriched in the aglycone form. These findings align with previous studies that link glycosylated phytoestrogens to enhanced thermoregulatory responses and metabolic stability [24]. Overall, these observations highlight the therapeutic potential of Rex and Rex-AG in managing key postmenopausal symptoms, particularly weight gain and vasomotor disturbances. The ability of these treatments to regulate body weight and stabilize thermoregulatory responses underscores their promise as natural, phytoestrogen-based alternatives to conventional estrogen therapies for postmenopausal health management.

### 2.4. Postmenopausal Biochemical Marker Profiles

Blood biochemical analysis in ovariectomized (OVX) rats provided insights into the systemic effects of estrogen depletion and the therapeutic potential of Rex and Rex-AG treatments. Estrogen (E2) levels were significantly reduced in the OVX group compared with the sham group, consistent with the known physiological impact of menopause-induced estrogen depletion (Table 2). Treatment with Rex and Rex-AG, however, led to a significant increase in E2 levels, suggesting that these extracts can partially restore estrogen levels, potentially through their phytoestrogenic properties [25,26,27]. In addition to estrogen, key biochemical markers associated with liver function and lipid metabolism were evaluated. Aspartate aminotransferase (AST), alkaline phosphatase (ALP), and triglyceride (TG) levels were significantly reduced in the E2-treated and Rex-treated groups compared with the OVX control group, reflecting improved physiological regulation post-treatment. These findings align with the established role of estrogen in maintaining liver function and lipid metabolism, which are often disrupted in postmenopausal states [28,29]. Among these markers, ALP levels were particularly informative, as elevated ALP is commonly linked to hepatic dysfunction, bone disorders, and certain cancers [30,31]. Elevated ALP is a hallmark biomarker of postmenopausal health deterioration and is often associated with estrogen depletion and bone turnover [32,33,34]. Notably, ALP levels in the Rex-treated group were comparable to those in the sham group, suggesting potential benefits for both bone health and liver protection. These findings underline the potential of Rex and Rex-AG to mitigate adverse effects associated with menopause by improving bone health and normalizing altered biochemical markers linked to estrogen depletion. The normalization of ALP levels, coupled with the observed improvements in other markers, offers promising therapeutic implications for managing menopausal symptoms. This study highlights Rex and Rex-AG as natural candidates for addressing menopausal health challenges, particularly in bone health and liver function. The results provide valuable insights into the physiological benefits of these compounds and emphasize the need for further clinical exploration to validate their potential therapeutic applications.

### 2.5. Impact on Vaginal Epithelial Keratinization

**Figure 4:**
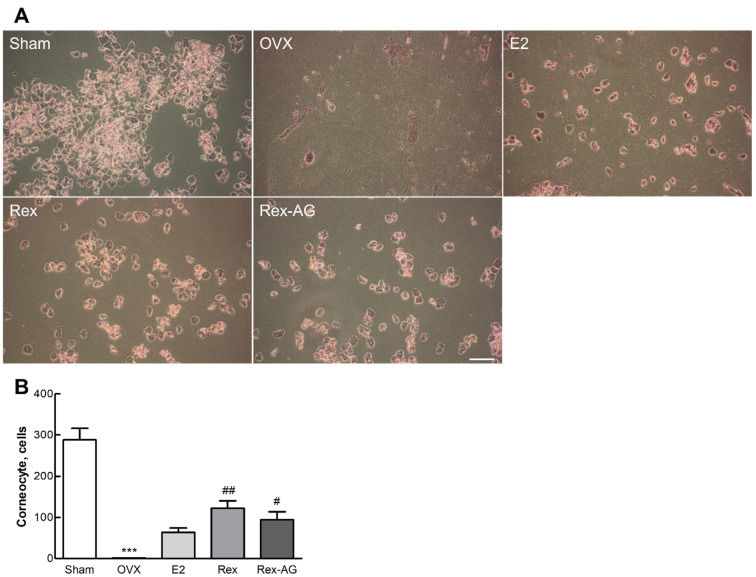
Analysis of vaginal keratinization in ovariectomized (OVX) rats. Rats were administered 150 mg/kg/day of Rex or Rex-AG for six weeks, with 17β-estradiol (E2) as a positive control. (**A**) Representative images of a Giemsa-stained vaginal epithelial swab. (**B**) Quantitative analysis of corneocyte counts. The scale bar in the image represents 100 µm. Data are presented as the mean ± standard deviation (n = 7). Statistical significance is indicated by *** *p* < 0.001 compared with the sham group or # *p* < 0.05 and ## *p* < 0.01 compared with the OVX group. Rex, *Styphnolobium japonicum* L. fruit extract (glycoside form); Rex-AG, Rex aglycone form.

The effectiveness of Rex and Rex-AG in alleviating postmenopausal vaginal symptoms, particularly vaginal dryness and keratinization, was evaluated in ovariectomized (OVX) Sprague–Dawley (SD) rats. Vaginal dryness and keratinization are prevalent complications in postmenopausal women due to estrogen depletion, leading to reduced mucosal flexibility and discomfort [35]. To assess these changes, Giemsa staining was used to differentiate between nucleated and cornified cells in the vaginal epithelium, a well-established indicator of estrogenic activity in the vaginal mucosa [36]. The results showed a significant increase in the proportion of cornified cells in the Rex and Rex-AG treatment groups, comparable to that in the estrogen-supplemented positive-control group (Figure 4). In contrast, the OVX control group exhibited markedly lower levels of cornified cells, reflecting the detrimental effects of estrogen deprivation on the vaginal epithelium. These findings indicate that both Rex and Rex-AG mimic estrogen-like effects, promoting epithelial health and reducing vaginal keratinization. This reduction suggests improved mucosal flexibility and hydration, critical factors in alleviating postmenopausal vaginal symptoms [37]. These findings are particularly significant in addressing postmenopausal vaginal symptoms such as dryness and keratinization, which are commonly reported yet difficult to treat effectively [38]. These results highlight the potential of Rex and Rex-AG as natural alternatives for managing vaginal symptoms associated with menopause. By simulating estrogenic effects in the vaginal epithelium, these treatments may improve vaginal hydration, flexibility, and overall comfort. This study underscores the importance of developing functional food products or natural therapies that target challenging postmenopausal symptoms such as vaginal dryness and keratinization. Future clinical research is warranted to validate these findings and to optimize dosing strategies for human application.

### 2.6. Morphological Changes in Uterine and Adipose Tissues

**Figure 5:**
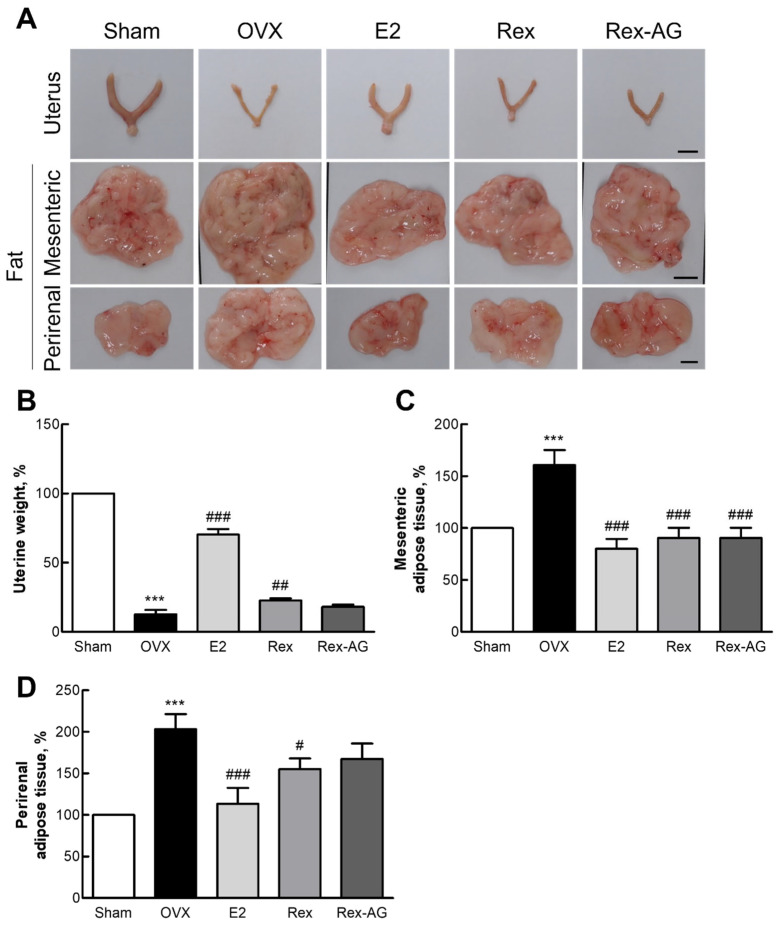
Comparison of uterine and adipose tissues in ovariectomized (OVX) rats. Rats were administered 150 mg/kg/day of Rex or Rex-AG for six weeks, with 17β-estradiol (E2) as a positive control. (**A**) Representative images of the uterus, mesenteric fat, and perirenal fat, illustrating the morphological effects of the treatments. (**B**–**D**) Relative tissue masses of the uterus (**B**), mesenteric fat (**C**), and perirenal fat (**D**). Scale bars in images correspond to 1 cm. Data are presented as the mean ± standard deviation (n = 7). Statistical significance is indicated by *** *p* < 0.001 compared with the sham group or # *p* < 0.05, ## *p* < 0.01, and ### *p* < 0.001 compared with the OVX group. Rex, *Styphnolobium japonicum* L. fruit extract (glycoside form); Rex-AG, Rex aglycone form.

The therapeutic potential of Rex and Rex-AG for managing postmenopausal symptoms was evaluated in OVX rats, with the key findings highlighting their safety and efficacy. At a daily dose of 150 mg/kg, both Rex and Rex-AG exhibited a favorable safety profile, as evidenced by the absence of significant changes in organ weights, indicating minimal risk of adverse organ-related effects (Supplementary Figure S3). One of the most notable findings was the increase in uterine weight in the Rex-treated group, which was significantly higher than that in the OVX control group (Figure 5A,B). This suggests that Rex possesses estrogen-like properties in effectively mitigating uterine atrophy—a hallmark symptom of postmenopausal estrogen depletion [38,39]. The observed effects emphasize the ability of Rex to mimic estrogen’s physiological role in maintaining uterine health and structure, a critical factor in overall postmenopausal health.

In addition to uterine health, significant reductions in perirenal and mesenteric fat weights were observed in both the Rex- and Rex-AG-treated groups, with Rex demonstrating a more pronounced effect (Figure 5C,D). These results align with the well-documented pattern of adipose tissue redistribution during menopause, which often leads to increased central adiposity and heightened metabolic risks [40,41]. The reduction in adipose tissue weights suggests that Rex, and to a lesser extent Rex-AG, may help counteract postmenopausal metabolic shifts by modulating fat deposition patterns.

These findings collectively demonstrate the therapeutic potential of Rex and Rex-AG in managing key postmenopausal symptoms. By reducing uterine atrophy and mitigating adipose tissue redistribution, Rex demonstrates dual benefits in improving both reproductive and metabolic health in postmenopausal states. These results not only highlight the promise of Rex and Rex-AG as natural alternatives for postmenopausal symptom management but also emphasize their potential role in enhancing the overall quality of life for postmenopausal women. Further clinical studies are warranted to validate these findings and to explore their mechanisms of action in human populations.

### 2.7. Adipose Tissue Gene Expression Alterations

**Figure 6:**
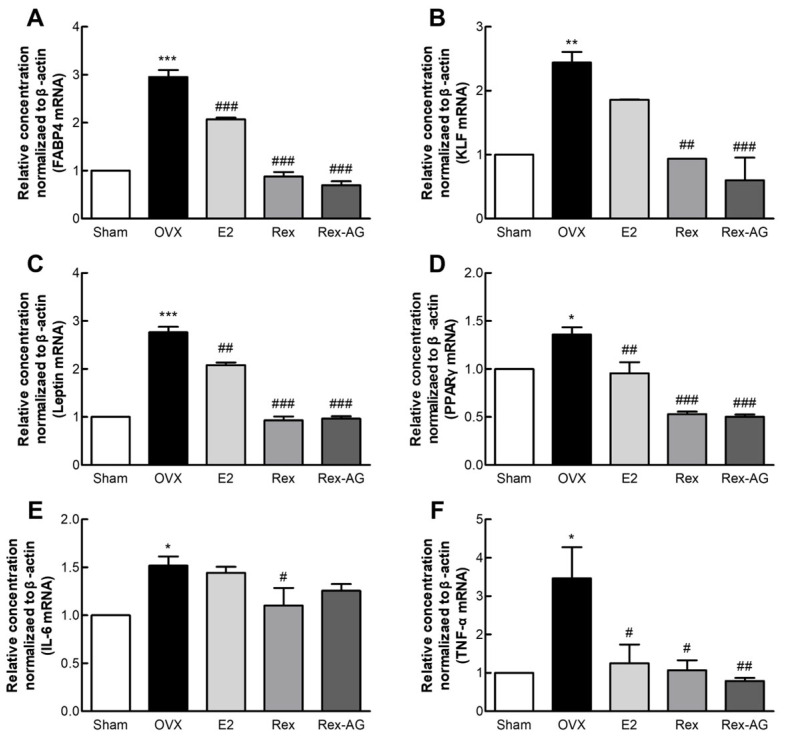
Gene expression analysis in mesenteric fat tissue of ovariectomized (OVX) rats. Rats were administered 150 mg/kg/day of Rex or Rex-AG for six weeks, with 17β-estradiol (E2) as a positive control. Relative expression levels of key metabolic and inflammatory genes were analyzed by qPCR: FABP4 (**A**), KLF (**B**), leptin (**C**), PPARγ (**D**), IL-6 (**E**), and TNF-α (**F**). Data are presented as the mean ± standard deviation (n = 7). Statistical significance is indicated as * *p* < 0.05, ** *p* < 0.01, and *** *p* < 0.001 compared with the sham group or # *p* < 0.05, ## *p* < 0.01, and ### *p* < 0.001 compared with the OVX group. Rex, *Styphnolobium japonicum* L. fruit extract (glycoside form); Rex-AG, Rex aglycone form; FABP4, fatty acid-binding protein 4; KLF, Krüppel-like factor; PPARγ, peroxisome proliferator-activated receptor γ; IL-6, interleukin 6; TNF-α, tumor necrosis factor α.

This study explored the effects of Rex and Rex-AG on gene expression in perirenal adipose tissues of postmenopausal OVX rats, revealing significant alterations that underscore their potential efficacy in managing menopause-associated obesity and metabolic dysfunctions. Both Rex and Rex-AG treatments resulted in the marked downregulation of key genes implicated in adipogenesis and obesity, including fatty acid-binding protein 4 (FABP4), Krüppel-like factors (KLFs), leptin, and peroxisome proliferator-activated receptor γ (PPARγ), compared with the untreated OVX control group. Notably, the reductions observed in the Rex and Rex-AG groups were more pronounced than those in the E2-treated positive-control group (Figure 6A–D). These findings are particularly significant given the critical roles of these genes in postmenopausal weight gain and adipose tissue dysfunction. FABP4 plays a pivotal role in fatty acid storage and adipocyte differentiation, contributing to fat accumulation in adipose tissues [42,43]. Similarly, leptin, which regulates energy homeostasis, can promote leptin resistance, a hallmark of obesity, when overexpressed [44]. PPARγ, a master regulator of adipocyte differentiation, is central to the pathogenesis of obesity and related metabolic disorders [45]. The suppression of these genes by Rex and Rex-AG indicates their potential to counteract adipogenic pathways associated with menopause.

In addition to modulating adipogenic gene expression, Rex and Rex-AG significantly suppressed the expression of pro-inflammatory cytokines, including interleukin 6 (IL-6) and tumor necrosis factor α (TNF-α) (Figure 6E,F). Chronic low-grade inflammation, characterized by elevated levels of IL-6 and TNF-α, is a well-documented consequence of increased fat accumulation in postmenopausal women [46,47]. These cytokines contribute to systemic inflammation, insulin resistance, and metabolic dysfunctions, exacerbating the risks associated with obesity during menopause.

Collectively, these findings highlight the dual benefits of Rex and Rex-AG in mitigating menopause-associated metabolic disturbances. By downregulating adipogenic and pro-inflammatory gene expression, these treatments demonstrate potential for alleviating obesity and reducing chronic inflammation, two interrelated challenges faced by postmenopausal women. These results position Rex and Rex-AG as promising therapeutic agents for managing postmenopausal symptoms, particularly those related to weight gain, adipose tissue dysfunction, and inflammation. Further clinical studies are warranted to validate these findings and to explore their broader applications in metabolic health management.

### 2.8. Bone Tissue Histomorphology and Molecular Gene Expression

**Figure 7:**
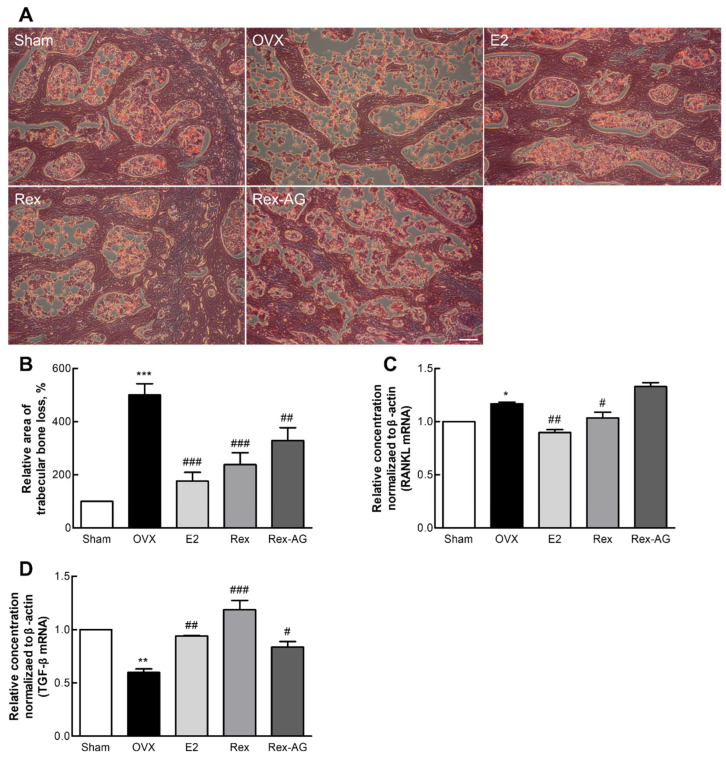
Evaluation of bone features and gene expression profiles in ovariectomized (OVX) rats. Rats were administered 150 mg/kg/day of Rex or Rex-AG for six weeks, with 17β-estradiol (E2) as a positive control. (**A**) Representative hematoxylin-and-eosin-stained transverse sections of femoral bone tissues. (**B**) Quantification of the spongy bone loss area using ImageJ software (version 1.47). (**C**,**D**) Relative expression levels of the RANKL (**C**) and TGF-β (**D**) genes in bone marrow cells as analyzed by qPCR. Scale bars in the images correspond to 100 µm. Data are presented as the mean ± standard deviation (n = 7). Statistical significance is indicated by * *p* < 0.05, ** *p* < 0.01 and *** *p* < 0.001 compared with the sham group or # *p* < 0.05, ## *p* < 0.01, and ### *p* < 0.001 compared with the OVX group. Rex, *S. japonicum* L. fruit extract (glycoside form); Rex-AG, Rex aglycone form; RANKL, receptor activator of nuclear factor-kappa B ligand; TGF-β, transforming growth factor β.

This study evaluated the therapeutic potential of Rex and Rex-AG in managing postmenopausal osteoporosis by analyzing both the histological morphology and gene expression in femoral bone tissues of OVX rats. Histological examination revealed that both Rex and Rex-AG treatments effectively preserved trabecular bone integrity, with the Rex-treated group showing bone density levels comparable to those in the estrogen-supplemented positive-control group (Figure 7A,B). This preservation highlights the ability of Rex to counteract the rapid bone loss commonly associated with postmenopausal estrogen deficiency. Notably, Rex demonstrated a more pronounced effect on bone integrity compared with Rex-AG, suggesting that the glycosylated form may have enhanced efficacy [48,49].

At the molecular level, the gene expression analysis focused on osteoporosis-related genes, including receptor activator of nuclear factor κB ligand (RANKL) and transforming growth factor β (TGF-β), which play pivotal roles in bone resorption and formation, respectively. Quantitative PCR (qPCR) analysis showed that Rex treatment significantly decreased RANKL expression while increasing TGF-β expression compared with the OVX control group (Figure 7C,D). These changes suggest a favorable modulation of bone remodeling pathways, as RANKL is a critical driver of osteoclast-mediated bone resorption, while TGF-β promotes osteoblast activity and bone regeneration [50,51]. The observed downregulation of RANKL and upregulation of TGF-β in the Rex-treated group align with its superior ability to preserve bone density and support bone regeneration compared with Rex-AG. These molecular changes complement the histological findings, collectively indicating that Rex promotes a shift toward bone preservation and repair, addressing both the structural and functional deficits of postmenopausal osteoporosis.

These dual effects—preservation of trabecular bone integrity and modulation of key genetic pathways—highlight Rex and Rex-AG as promising natural agents for managing osteoporosis. The ability of these treatments to mitigate bone loss and enhance bone regeneration underscores their potential as comprehensive therapeutic options for postmenopausal women. Further studies, particularly clinical trials, are needed to validate these findings and to explore optimal dosing strategies for maximizing their bone health benefits.

### 2.9. Systemic Genistein Absorption and Distribution

**Figure 8:**
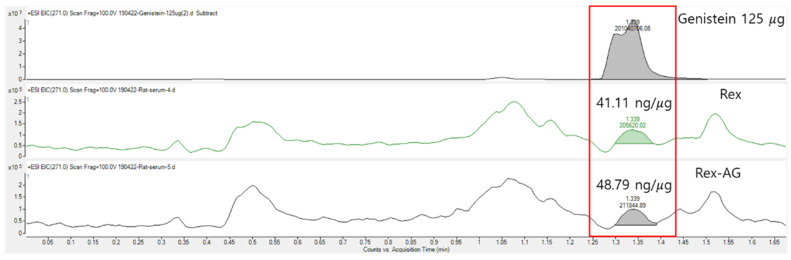
Representative LC-MS chromatograms showing genistein detection in serum samples of ovariectomized (OVX) rats. Chromatograms display the single-ion monitoring (SIM) of genistein (*m*/*z* = 271.0) in rat sera following six weeks of treatment with Rex or Rex-AG (150 mg/kg/day). The characteristic peak profile reflects the interaction of genistein with serum matrix components under the specific analytical conditions employed. For quantification, the total area of the observed peaks was integrated. The inset bar graph displays the mean serum genistein concentrations (ng/mL) determined from three independent samples per group (n = 3), with error bars representing the standard deviation. Serum genistein levels were 41.11 ± 5.81 ng/mL in the Rex group and 48.90 ± 10.74 ng/mL in the Rex-AG group. Rex, *S. japonicum* L. fruit extract (glycoside form); Rex-AG, Rex aglycone form.

To verify the systemic bioavailability of active compounds, serum genistein concentrations were quantified following six weeks of oral administration of Rex and Rex-AG. Blood genistein levels were measured at 41.11 ± 5.81 ng/mL in the Rex group and 48.90 ± 10.74 ng/mL in the Rex-AG group (Figure 8). These concentrations indicate efficient intestinal absorption and systemic bioavailability of genistein in its active aglycone form, which is essential for eliciting therapeutic effects. LC-MS analysis was conducted using the single-ion monitoring (SIM) mode with an *m*/*z* of 271.0 for genistein detection. The characteristic peak profile observed in the LC-MS chromatograms reflects the interaction of genistein with endogenous serum matrix components under the specific analytical conditions employed. For quantification purposes, the total area of the observed peak was integrated to maintain analytical consistency across all samples, with method validation confirming the acceptability of the precision and accuracy parameters. The presence of deglycosylated genistein in the bloodstream after administration of both Rex and Rex-AG confirms that effective metabolic processing and intestinal deglycosylation has occurred. This finding underscores the role of intestinal bacteria, such as *Lactobacillus plantarum*, in converting glycosylated compounds into their bioactive aglycone forms, as demonstrated in prior studies [21]. Despite the slightly higher genistein concentrations observed in the Rex-AG group, this difference is unlikely to result in a significant variation in therapeutic efficacy in addressing postmenopausal symptoms, including osteoporosis and adipose tissue changes [52]. Importantly, the data demonstrate that the glycosylated form (Rex) does not impair genistein absorption. Instead, its efficient deglycosylation ensures comparable bioavailability to that of Rex-AG, highlighting the versatility of both formulations in delivering bioactive genistein. This equivalence is particularly relevant as it suggests that Rex, which contains the glycosylated precursor, can serve as an effective therapeutic option while potentially providing sustained release of genistein due to its gradual conversion.

The efficient absorption and distribution profiles of genistein from both Rex and Rex-AG reinforce their therapeutic potential in managing postmenopausal health issues. By ensuring sufficient systemic levels of genistein, these treatments offer a robust basis for addressing estrogen-deficiency-related conditions. These findings lay a solid foundation for future clinical studies to explore the comparative efficacy of Rex and Rex-AG in improving postmenopausal symptoms, with a focus on optimizing dosing strategies and exploring long-term effects.

### 2.10. Comparison with Non-Hormonal Approaches for Postmenopausal Symptom Management

Recent advances in postmenopausal treatment options have expanded beyond traditional hormone replacement therapy (HRT) to include non-hormonal alternatives. Notably, fezolinetant, a neurokinin 3 receptor (NK3R) antagonist, was recently approved for the management of moderate to severe vasomotor symptoms associated with menopause. This novel drug represents a significant shift in the therapeutic approach as it targets the hypothalamic KNDy (kisspeptin, neurokinin B, and dynorphin) neuron pathway implicated in hot-flash generation rather than replacing depleted estrogen.

When considering treatment options for postmenopausal symptoms, both hormonal and non-hormonal approaches offer distinct advantages and limitations. Hormone-based therapies like traditional HRT and phytoestrogens (such as Rex and Rex-AG examined in this study) address the fundamental hormonal deficiency underlying menopause and potentially offer comprehensive benefits across multiple physiological systems, including for bone health, metabolic regulation, and vaginal atrophy. However, concerns about cancer risks and cardiovascular events with conventional HRT have driven the interest in non-HRT alternatives.

Non-hormonal approaches like fezolinetant demonstrate effectiveness against specific symptoms, particularly hot flashes, with potentially improved safety profiles regarding their cancer risk. However, they may not address the broader spectrum of menopausal changes across multiple organ systems.

The phytoestrogen-based approach investigated in our study potentially offers a middle ground—providing estrogen-like benefits for bone health, metabolism, and vaginal symptoms while potentially carrying fewer risks than conventional HRT due to their selective estrogen-receptor modulation. Future comparative studies examining Rex and Rex-AG treatments alongside non-hormonal options like fezolinetant would provide valuable insights into their relative efficacies for specific symptom clusters and help inform personalized treatment decisions for postmenopausal women.

## 3. Materials and Methods

### 3.1. Sample Preparation

*S. japonicum* L. fruit (Korean Food Code: B-GA013000) was sourced from NOVAREX (Osong, Chungbuk, Republic of Korea). The extract (Rex) was prepared via 60 ± 10% fermented ethanol (food-grade ethanol, CAS No. 64-17-5; Korea Ethanol Supplies Company, Seoul, Republic of Korea) reflux extraction, concentrated to 60% Brix, sterilized, and spray-dried into powder. Standard sophoricoside (≥98%) and genistein (≥98%) were obtained from Sigma-Aldrich (St. Louis, MO, USA). A *Lactobacillus plantarum* strain isolated from Prunus tomentosa, chosen for its rich natural microbiota and its ability to enhance probiotic fermentation efficiency, and other probiotics (*L. brevis*, *L. casei*, *L. fermentum*, and *B. subtilis*) were obtained from Korean culture collections. Growth media (MRS; De Man, Rogosa and Sharpe nutrient media) were used for strain cultivation. For biotransformation, Rex (4% Brix), selected based on previous studies as the optimal concentration for efficient microbial activity and glycoside conversion, was inoculated with *L. plantarum* (2.0 × 10^7^ CFU/mL) and fermented at 30 °C, 150 rpm for 24–96 h. After fermentation, the mixture was centrifuged (8000× *g*, 60 min), filtered, and lyophilized to obtain biotransformed Rex aglycone (Rex-AG), which exhibits enhanced bioavailability and increased enzymatic conversion efficiency compared with Rex.

### 3.2. High-Performance Liquid Chromatography (HPLC) Analysis

HPLC analysis was performed using a Thermo Fisher Scientific U3000 HPLC system (Thermo Fisher Scientific, Waltham, MA, USA) equipped with a diode-array detector. Chromatographic separation was achieved on a YMC Triart C18 column (250 mm × 4.6 mm, 5 μm particle size, 12 nm pore size; YMC Co., Ltd., Kyoto, Japan) maintained at 30 °C. The mobile phase consisted of distilled water (Solvent A) and acetonitrile (Solvent B). It was employed in a gradient elution program. For sophoricoside and genistein analysis, the gradient profile was set as follows: 0–3 min, 10% B; 3–45 min, linear gradient to 45% B; 45–46.5 min, ramp to 100% B; 46.5–50 min, hold at 100% B; 50–51.5 min, return to initial conditions (10% B). The flow rate was maintained at 1.0 mL/min, and detection was performed at a wavelength of 280 nm. For ginsenoside separation, the following gradient elution program was applied: 0–10 min (20% B), 10–40 min (20–32% B), 40–55 min (32–50% B), 55–70 min (50–65% B), 70–72 min (65–90% B), 72–82 min (90% B), 82–84 min (90–20% B), and 84–90 min (20% B). This program was conducted at a flow rate of 1.6 mL/min. Detection was achieved at 203 nm. The sample was prepared at a final concentration of 10 mg/mL and injected in a volume of 10 µL. Standard materials were prepared at various concentrations. Retention time and peak areas were logged using the Chromeleon software (version 7.2.10; Thermo Fisher Scientific). Each peak was identified by comparing its retention time and ultraviolet (UV) spectra with those of standards. The flow rate was maintained at 1.0 mL/min, and detection was performed at a wavelength of 280 nm.

### 3.3. Liquid Chromatography–Mass Spectrometry (LC-MS) Quantification of Genistein

LC-MS was used to measure the amount of genistein in blood. Serum was obtained from blood using Vacutainer SST II Plus plastic serum tubes (BD 367955; BD Biosciences, Franklin Lakes, NJ, USA). It was then mixed with cold methanol four times its volume and centrifuged at 10,000× *g* for 1 min. The resulting supernatant was then filtered using a 0.22 μm syringe filter. Analysis was performed using an Agilent 1290 Infinity HPLC system coupled with a 6470 Triple Quadrupole MSD (Agilent Technologies, Santa Clara, CA, USA). Chromatographic separation was achieved with an EclipsePlus C18 column (50 mm × 2.1 mm, 1.8 μm particle size; Agilent Technologies). The mobile phase consisted of distilled water (Solvent A) and acetonitrile (Solvent B). It was used with the following gradient elution program: initial conditions at 20% B, increased to 80% B over 1 min, holding for 2.5 min, and then returned to 20% B. The flow rate was set at 0.4 mL/min with an injection volume of 2 μL. The column temperature was maintained at 30 °C. Quantification was based on a comparison of the peak area of the standard with that of the sample. Detection was performed using electrospray ionization (ESI) in the positive-ion mode. Genistein content was analyzed in the single-ion monitoring (SIM) mode with an *m*/*z* of 271.0 (Supplementary Figure S1).

The SIM mode was selected for genistein quantification following preliminary method development that demonstrated adequate sensitivity, selectivity, and linear response for the concentration ranges expected in our serum samples. While the multiple-reaction monitoring (MRM) mode could potentially offer enhanced specificity and sensitivity, particularly for complex biological matrices, the SIM approach provided sufficient analytical performance for our specific experimental design and sample sets. While separate analytical methods were employed for biotransformation monitoring (HPLC) and serum genistein quantification (LC-MS) in this study, future investigations could benefit from a unified LC-MS/MS approach. A harmonized analytical protocol could potentially improve cross-matrix comparability between plant extracts and biological samples, enhance the sensitivity for low-abundance metabolites, and streamline the analytical workflow. However, the current separation methods were selected based on the distinct sample matrices (plant extract vs. blood serum) and the significantly different concentration ranges of the analytes in each matrix, which allow for optimized sensitivity and specificity in each application. Method validation confirmed that acceptable precision, accuracy, and recovery rates for genistein quantification were achieved under these conditions.

### 3.4. Experimental Animal Model and Ovariectomy Procedure

Female Sprague–Dawley (SD) rats (12 weeks old, 220–240 g) were acquired from KOATECH (Gyeonggi, Republic of Korea) and housed under controlled conditions (temperature of 21 ± 2 °C, humidity of 40–60%, and a 12 h/12 h light/dark cycle). After a week of acclimatization with approval from the Gangneung–Wonju National University Animal Care and Use Committee (GWNU-2018-21, approved on 17 September 2018), rats were randomly divided into five groups: sham-operated (Sham), ovariectomized control (OVX), OVX treated with 100 μg/kg/day of 17β-estradiol (OVX + E2), OVX treated with 150 mg/kg/day of Rex (OVX + Rex), and OVX treated with 150 mg/kg/day of Rex-AG (OVX + Rex-AG). After another week of acclimation, ovariectomy was performed for selected rats, which involved dorsal fur shaving, back incisions, bilateral ovary removal, and suturing [53]. The sham group underwent a similar procedure but without ovary removal. Postoperative monitoring was carried out to ensure recovery and the absence of complications such as incision rupture or inflammation. Out of the 50 animals operated on, only those that fully recovered were included in the subsequent experimental analyses (n = 7). After recovery, rats were fed diets formulated according to their group for a total of six weeks. Sham and OVX groups received, ad libitum, the AIN-76A phytoestrogen-free diet (Harlan Teklad, Madison, WI, USA), while treatment groups received a custom-manufactured diet mixed with their respective treatments (Daehan Biolink, Chungbuk, Republic of Korea). Body weight was measured twice a week at consistent times.

### 3.5. Assessment of Vasomotor Symptoms

Vasomotor symptoms commonly manifest as initial symptoms in postmenopausal women. They are predominantly characterized by hot flashes, in which the skin on the face, neck, and chest suddenly turns red, accompanied by an unpleasant sensation of heat and sweating throughout the body [54]. To observe vasomotor symptoms in our study, temperatures of rats were measured two days before autopsy. Rectal temperatures were recorded at 10 min intervals for 120 min immediately following a 15 min forced running session on a motorized treadmill (MK-680S; Muromachi Kikai, Tokyo, Japan) at a speed of 15 m/min. The change in rectal temperature was calculated as a percentage based on each animal’s baseline temperature, which was the average temperature measured 20 min before the forced running session. To minimize variability, rectal temperatures were recorded using an electronic thermometer (MT200; Microlife, Taipei, Taiwan).

### 3.6. Evaluation of Vaginal Epithelial Cell Changes (Vagina Cornification)

To assess vaginal epithelial changes, cells were collected from vaginal walls of rats in each group using a sterile swab immediately before autopsy. These cells were spread onto a glass slide and subjected to Giemsa staining. The numbers of nucleated and cornified cells were counted to determine the proportion of cornified epithelial cells. In this model, estrogen influences vaginal epithelial cornification, with higher cornification associated with normal estrogenic activity and lower cornification typically seen in estrogen-deficient states like menopause [55]. Therefore, an increased proportion of cornified cells indicates improved vaginal epithelial health and reduced vaginal dryness.

### 3.7. Tissue Morphology and Adipose Tissue Analysis

Organs were collected to evaluate potential toxicity and treatment effects on tissue morphology. Prior to autopsy, animals were fasted for 12 h and then anesthetized using ether. Serum was separated from arterial blood and stored at −80 °C for subsequent hormone and biochemical parameter analyses. During autopsy, various tissues, including the liver, spleen, kidneys, uterus, perirenal fat, mesenteric fat, and femur bones (left and right distinguished), were excised. Tissue weight was measured to assess potential organ-specific effects or abnormalities related to treatment. The right femurs were fixed in 10% neutral buffered formalin solution for bone loss analysis.

### 3.8. Hormonal and Biochemical Parameter Measurements

To assess hormonal and biochemical status, blood samples were collected and processed to obtain serum by centrifugation (3000× *g*, 15 min, 4 °C). For hormonal analysis, the concentration of serum E2 was quantitatively determined using an enzyme-linked immunosorbent assay (ELISA) kit (Cayman Chemical, Ann Arbor, MI, USA) following the manufacturer’s protocol, which included appropriate sample dilution and incubation steps. The assay had a detection sensitivity appropriate for the expected concentration range in our experimental model. Comprehensive biochemical analyses were conducted to evaluate serum markers indicative of metabolic and liver functions. These included calcium (Ca), using the o-cresolphthalein complexone method; aspartate aminotransferase (AST), using the IFCC reference method; alkaline phosphatase (ALP), using the p-nitrophenyl phosphate method; and triglyceride (TG), using the GPO-PAP method. All biochemical measurements were performed using an automatic analyzer (INTEGRA 400, Roche, Mannheim, Germany), with appropriate calibration and quality controls to ensure analytical accuracy and precision.

### 3.9. Gene Expression Analysis in Bone and Adipose Tissues

We conducted gene expression analyses for both femur and adipose tissues extracted from an OVX model to gain insights into their respective roles and interactions under conditions of estrogen depletion. After a six-week administration period, rats were euthanized by ether inhalation. Their femurs and a portion of the mesenteric adipose tissue were excised. Bone marrow cells were isolated from the left femur for evaluating gene expression [56]. This process involved halving the femurs and placing one of the halves into a microcentrifuge tube. Bone marrow was extracted by centrifugation at 5000× *g* for 5 min. Cells were then filtered through a 40 µm cell strainer. Erythrocytes were lysed using a red blood cell (RBC) lysis buffer (BioLegend, San Diego, CA, USA). The adipose tissue was crushed and powdered in liquid nitrogen, followed by homogenization for 1 min using an Ultratorax homogenizer (IKA Labortechnik, Staufen, Germany). RNA was extracted from both tissues and used for cDNA synthesis and quantitative real-time polymerase chain reaction (qPCR) to analyze the expression levels of genes related to bone and adipose tissue metabolism and health.

### 3.10. Quantitative Real-Time Polymerase Chain Reaction (qPCR) Analysis

Total RNA was extracted from selected tissues (femur and adipose tissue) using TRIzol reagent (Thermo Fisher Scientific) according to the manufacturer’s instructions. For complementary DNA (cDNA) synthesis, 1 µg of total RNA was used as a template. cDNA was synthesized using the ImProm-II Reverse Transcriptase System (Promega, Madison, WI, USA) following the manufacturer’s protocol. This process included mixing the RNA with an oligo dT primer and deoxynucleotide mix, followed by the addition of reverse transcriptase and incubation at 42 °C for 1 h and then at 70 °C for 15 min to terminate the reaction. qPCR reactions were prepared using the SensiMixTM SYBR Hi-ROX PCR Master Mix (Bioline, London, UK) in a total volume of 10 µL, which included primers specific for the target genes (Supplementary Table S1). Amplification and detection were performed using a Rotor-Gene 6000 real-time PCR system (Qiagen, Hilden, Germany). Cycling conditions were as follows: initial denaturation at 95 °C for 15 min, followed by 45 cycles of denaturation at 95 °C for 15 s, annealing at 52 °C for 15 s, and extension at 72 °C for 10 s. Relative expression levels of the target genes were calculated using the 2^−ΔΔCt^ method, with β-actin serving as an internal control for data normalization [53]. Results are expressed as the fold-change relative to the sham group.

### 3.11. Histomorphometric Analysis of Trabecular Bone

For microscopic analysis, right femurs were decalcified using 10% ethylenediaminetetraacetic acid (EDTA) at 4 °C for 14 days. After decalcification, femurs were processed for paraffin embedding, and sections with a thickness of 5 µm were prepared. These sections were then stained with hematoxylin and eosin (H&E) to visualize the bone microarchitecture. The extent of trabecular bone loss was quantitatively assessed using ImageJ software (National Institutes of Health, Bethesda, MD, USA). This analysis involved measuring the trabecular area as a percentage of the total bone area [(trabecular area/total bone area) × 100%] to quantify the bone loss.

### 3.12. Statistical Analysis

Data are presented as the mean ± standard deviation. The statistical significance of differences between two groups was analyzed using the Student’s *t*-test, while comparisons among multiple groups were performed using one-way analysis of variance (ANOVA), followed by Tukey’s post hoc test. All statistical analyses were conducted using GraphPad Prism Version 5.01 (GraphPad Software Inc., San Diego, CA, USA). A *p*-value less than 0.05 (*p* < 0.05) was considered statistically significant. Throughout the manuscript, significance symbols are used consistently as follows: asterisks (* *p* < 0.05, ** *p* < 0.01, *** *p* < 0.001) indicate significant differences compared with the sham group, while hash signs (# *p* < 0.05, ## *p* < 0.01, ### *p* < 0.001) indicate significant differences compared with the OVX control group.

## 4. Conclusions

This study highlights the potential of Rex and Rex-AG as promising therapeutic agents for managing postmenopausal symptoms. Both compounds effectively mitigated estrogen-depletion-related symptoms in OVX SD rats, demonstrating comprehensive therapeutic potential across multiple physiological domains, including in terms of osteoporosis, metabolic changes, and vasomotor and vaginal symptoms.

The key findings revealed the bioavailability of genistein, with effective absorption and systemic distribution observed in both Rex and Rex-AG treated groups. The serum genistein concentrations (41.11 ± 5.81 ng/mL in Rex and 48.90 ± 10.74 ng/mL in Rex-AG) achieved in our study confirm the successful intestinal absorption and biotransformation of these compounds. The natural biotransformation process occurring in the gut microbiome effectively converts sophoricoside to genistein, eliminating the need for additional fermentation or enzymatic processing. This suggests that the current extraction process can be directly utilized without complex additional treatments.

Future research could explore strategies to further enhance the bioavailability of these compounds, as improved absorption might potentially increase their therapeutic efficacy. The therapeutic benefits were evident across multiple physiological systems. Both compounds improved bone density by regulating osteoporosis-related genes, such as downregulating RANKL and upregulating TGF-β, thus preserving bone integrity. Additionally, Rex and Rex-AG normalized key markers of liver function and lipid metabolism, including AST, ALP, and TG levels, indicating potential hepatoprotective effects. Beyond bone and liver health, the compounds demonstrated significant potential in body weight regulation, adipose tissue reduction, and the suppression of pro-inflammatory cytokines like IL-6 and TNF-α. These findings position Rex and Rex-AG as multifaceted alternatives to conventional HRT, offering a natural approach to managing postmenopausal metabolic shifts and systemic inflammation.

The ability to use the extract in its native form, relying on the body’s natural metabolic pathways, represents a significant advancement in developing accessible and effective natural alternatives to conventional HRT. However, further research, including extensive clinical trials and long-term studies, is needed to validate their safety and efficacy in human populations. In conclusion, Rex and Rex-AG represent significant advancements as phytoestrogenic agents. Their practicality, accessibility, and potential efficacy as natural treatment options pave the way for developing innovative solutions that offer hope for an improved quality of life for postmenopausal women.

## Figures and Tables

**Table 1:** Screening of bacterial strains for their deglycosylation efficiency. Comparative analysis of deglycosylation levels induced by *L. plantarum* and *B. subtilis* during the biotransformation of sophoricoside to genistein. Rg3, a well-studied ginsenoside, was included as a reference compound to benchmark the bioconversion efficiency. PAE (*P. ginseng* extract), a rich source of glycoside saponins, was used as the substrate for evaluating the bacterial biotransformation performance. Deglycosylation efficiency was quantified by changes in the sophoricoside, genistein, and Rg3 content, which was analyzed via HPLC (see Supplementary Figure S2).

Subject	Fermentation	Rg3 Amount (mg/g)	Increase Ratio Compared with PAE
*Panax ginseng* extract (PAE)	–	0.15	–
	*L. plantarum*	1.15	7.6
	*B. subtilis*	0.87	5.8

**Table 2:** Comparative blood hormonal and biochemical analysis in OVX rats administered with Rex and Rex-AG. OVX rats were administered 150 mg/kg/day of either Rex or Rex-AG for six weeks, with 17β-estradiol (E2) serving as a positive control. Data are presented as the mean ± standard deviation (n = 4). Statistical significance is indicated as ** *p* < 0.01 and *** *p* < 0.001 compared with the sham group or ^#^ *p* < 0.05, ^##^ *p* < 0.01, and ^###^ *p* < 0.001 compared with the OVX group. Rex, *S. japonicum* L. fruit extract (glycoside form); Rex-AG, Rex aglycone form; Ca, calcium; AST, aspartate aminotransferase; ALP, alkaline phosphatase; TG, triglycerides.

Parameters	Sham	OVX	+E2	+Rex	+Rex-AG
E2 (pg/mL)	35.53 ± 3.78	14.99 ± 1.54 ***	35.86 ± 2.32 ^###^	29.51 ± 2.32 ^##^	25.67 ± 3.67 ^#^
Ca (mg/dL)	9.9 ± 0.24	9.7 ± 0.08	10.0 ± 0.24	9.9 ± 0.08	9.9 ± 0.08
AST (U/L)	141.45 ± 12.29	290.05 ± 39.4 **	131.05 ± 26.01 ^###^	98.75 ± 6.9 ^###^	190.25 ± 30.74 ^#^
ALP (U/L)	45.05 ± 0.12	69.00 ± 4.33 ***	51.85 ± 2.9 ^##^	45.60 ± 6.12 ^##^	62.10 ± 3.59
TG (mg/dL)	19.50 ± 4.08	48.65 ± 5.10 ***	54.30 ± 1.96	21.40 ± 0.41 ^###^	30.25 ± 4.78 ^##^

## Data Availability

The data that support the findings of this study are available from the corresponding author upon reasonable request.

## Supplementary Materials

The following supporting information can be downloaded at: https://www.mdpi.com/article/10.3390/molecules30102218/s1, Table S1: Primers for gene expression analysis; Figure S1: Mass spectrometry (MS) spectrum of the genistein standard showing the characteristic *m*/*z* value of 271.0 in the positive-ion mode used for identification and quantification; Figure S2: Bacterial deglycosylation and biotransformation in *Panax ginseng* extract (PAE). HPLC analysis was performed at a detection wavelength of 280 nm. Minor variations in retention times between the chromatograms result from slight differences in the column temperature and mobile-phase composition between analytical runs, which are within acceptable limits for HPLC analysis and do not affect the compound identification reliability; Figure S3: Organ tissue safety assessment in ovariectomized (OVX) rats, with the statistical analysis confirming no significant adverse effects on organ weights across treatment groups.

## Abbreviations

HRT	hormone replacement therapy
OVX	ovariectomy
Rex	*Styphnolobium japonicum* L. fruit extract
Rex-AG	Rex aglycone form
E2	17β-estradiol
PAE	*Panax ginseng* extract
HPLC	high-performance liquid chromatography
LC-MS	liquid chromatography–mass spectrometry
SD rat	Sprague–Dawley rat
ELISA	enzyme-linked immunosorbent assay
AST	aspartate aminotransferase
ALP	alkaline phosphatase
TG	triglyceride
qPCR	quantitative real-time polymerase chain reaction
cDNA	complementary DNA
EDTA	ethylenediaminetetraacetic acid
H&E	hematoxylin and eosin
ANOVA	analysis of variance
FABP4	fatty acid-binding protein 4
KLF	Krüppel-like factor
PPARγ	peroxisome proliferator-activated receptor γ
IL-6	interleukin 6
TNF-α	tumor necrosis factor α
RANKL	receptor activator of nuclear factor κB ligand
TGF-β	transforming growth factor β
