# Supplementary material for: Therapeutic Advantages of Isoflavone Glycoside and Aglycone Forms of Sophoricoside in the Amelioration of Postmenopausal Symptoms: Bone Health, Metabolic Regulation, and Systemic Inflammation
Source: Molecules. 2025 May 20;30(10):2218. doi: 10.3390/molecules30102218 (PMC12113653; doi:10.3390/molecules30102218)

Therapeutic Advantages of Isoflavone Glycoside and Aglycone Forms of Sophoricoside in the Amelioration of Postmenopausal Symptoms: Bone Health, Metabolic Regulation, and Systemic Inflammation

Jeong-Won Ahn [1,†], Hyun-Soo Kim [1,†], Kongara Damodar [2], Hee-Hyun Shin [3], Kyung-Mi Kim [3], Jung-Youl Park [4], Yeong-Min Yoo [5], Jae-Chul Jung [3] and Seong-Soo Joo [1,2,*]

[1] Department of Marine Bioscience, College of Life Science, Gangneung-Wonju National University, Gangneung 25457, Gangwon, Republic of Korea; 0000@gwnu.ac.kr (J.-W.A.); gustn4609@gwnu.ac.kr (H.-S.K.)

[2] Huscion MAJIC R&D Center, 331 Pangyo-ro, Seongnam 13488, Gyeonggi, Republic of Korea; kongaradamu@gwnu.ac.kr

[3] Life Science Research Institute, NOVAREX Co., Ltd., Cheongju 28220, Chungbuk, Republic of Korea; hhshin@novarex.co.kr (H.-H.S.); kkm3507@novarex.co.kr (K.-M.K.); jcjung@novarex.co.kr (J.-C.J.)

[4] Glocal University Project Group, Andong National University, 1375 Gyeongdong-ro, Andong 36729, Gyeongbuk, Republic of Korea; jypark09@anu.ac.kr

[5] Environmental Research Institute, Kangwon National University, Chuncheon-si 24341, Gangwon, Republic of Korea; yyeongm@hanmail.net

* Correspondence: ssj66@gwnu.ac.kr; Tel.: +82-33-640-2856

† These authors contributed equally to this work.

Supplementary Table S1. Primers for gene expression analysis

Gene	Direction	Sequence (5' to 3')	Accession
RANKL	Forward	5’-CAGCATCGCTCTGTTCCTGT	NM_011577.2
	Reverse	5’-CCAGAGTCGAGTCCTGCAAA	
TGF-β	Forward	5’-GGAGACGGAATACAGGGCTT	NM_011577.2
	Reverse	5’-GGTCCCAGACAGAAGTTGGC	
FABP4	Forward	5’-GTGGGAACCTGGAAGCTTGT	NM_024406.3
	Reverse	5’-TGCTCTTCACCTTCCTGTCG	
KLF	Forward	5’-GCCTTGTTGCAAGAGAACCA	NM_008453.5
	Reverse	5’-GCACCCATCATAGTCGCATC	
Leptin	Forward	5’-AGCTGCAAGGTGCAAGAAGA	NM_008493.3
	Reverse	5’-ACCGACTGCGTGTGTGAAAT	
PPARγ	Forward	5’-GGGAGTTCCTCAAAAGCCTG	XM_006237009.3
	Reverse	5’-GCAGCAGGTTGTCTTCAATG	
IL-6	Forward	5’-CCTTCCTACCCCAACTTCCA	NM_012589.2
	Reverse	5’-AGCACACTAGGTTTGCCGAG	
TNF-α	Forward	5’-GATTATGGCTCAGGGTCCAA	NM_013693
	Reverse	5’-GAGACAGAGGCAACCTGACC	
β-actin	Forward	5’-CATCAAAGAGAAGCTGTGCT	NM_001101.4
	Reverse	5’-GAAGGAAGGCTGGAAAAGAG	

Note: RANKL, receptor activator of nuclear factor-kappa B ligand; TGF-β, transforming growth factor β; FABP4, fatty acid binding protein 4; KLF, Krüppel-like factors; PPARγ, proliferator-activated receptor γ; IL-6, interleukin 6; TNF-α, tumor necrosis factor α.

Supplementary Figure S1. Liquid chromatography-mass spectrometry analysis of genistein standard. The analysis utilized Selected Ion Monitoring (SIM) in the Electrospray Ionization (ESI) positive ion mode, a technique effective for the precise detection of specific ions. The resulting mass-to-charge (m/z) value of 271.0 is indicative of the genistein molecule, confirming its presence and purity in the standard used for comparison in the study.

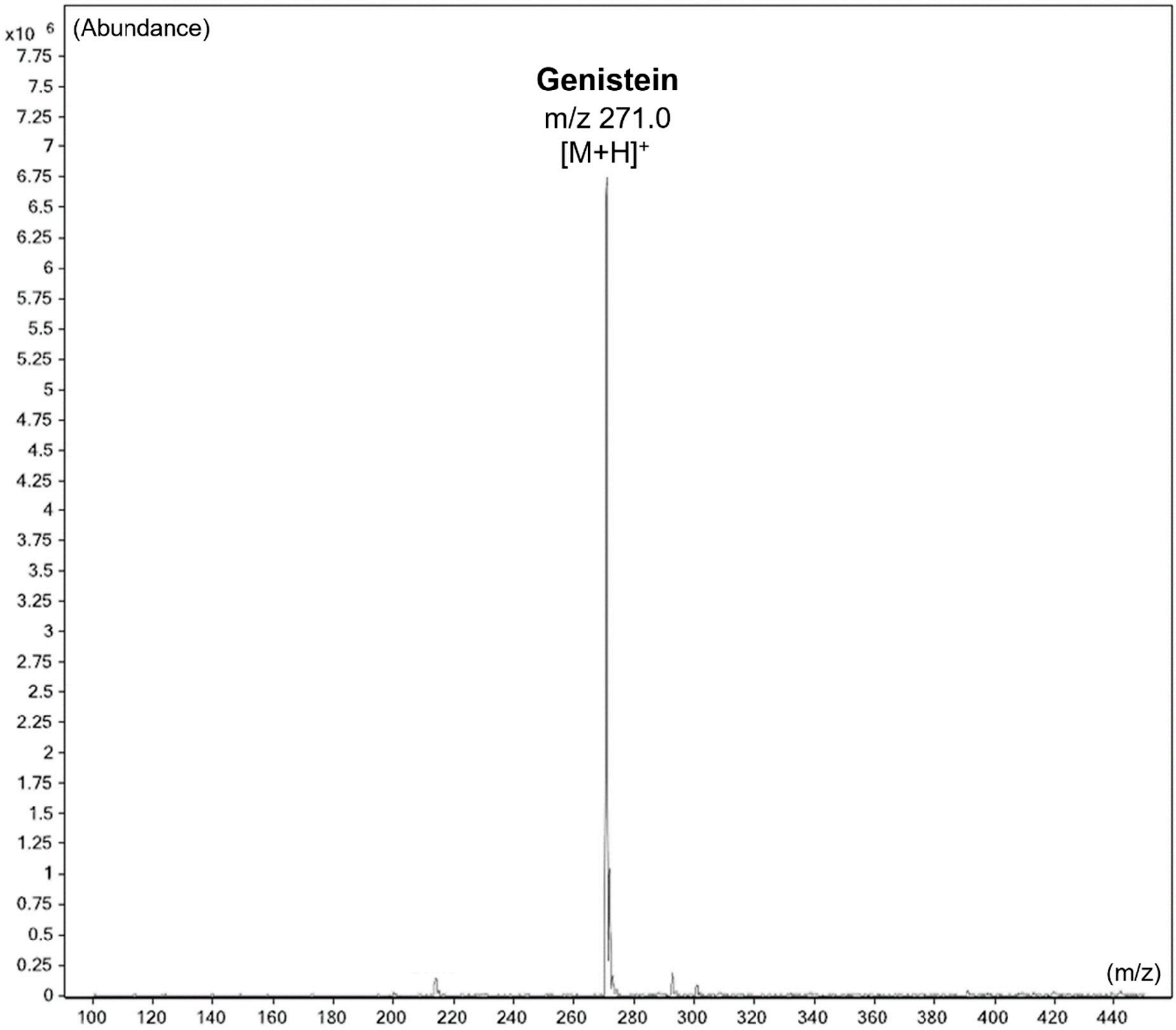

Supplementary Figure S2. Bacterial deglycosylation and biotransformation in *Panax ginseng* extract (PAE). HPLC analysis was performed at a detection wavelength of 280 nm to monitor the biotransformation of glycosides. This figure depicts the effects of *Lactobacillus plantarum* and *Bacillus subtilis* on deglycosylating ginsenoside compounds in PAE. (A) Presents high-performance liquid chromatography (HPLC) chromatograms comparing PAE before and after a 48-hour fermentation with the bacteria, illustrating the biotransformation process. (B and C) Show magnified sections of the chromatogram, highlighting the peaks corresponding to ginsenoside Rb2, Rd, and the transformed Rg3, demonstrating the effective conversion by the bacteria. (D) Provides a schematic representation of the biotransformation pathway through deglycosylation by these biocompatible bacteria. The diagram includes the structural changes from Rb2 and Rd to Rg3, with Arap (α-L-arabionopyranosyl) and Glc (β-D-glucopyranosyl) indicating specific sugar moieties involved in the process.

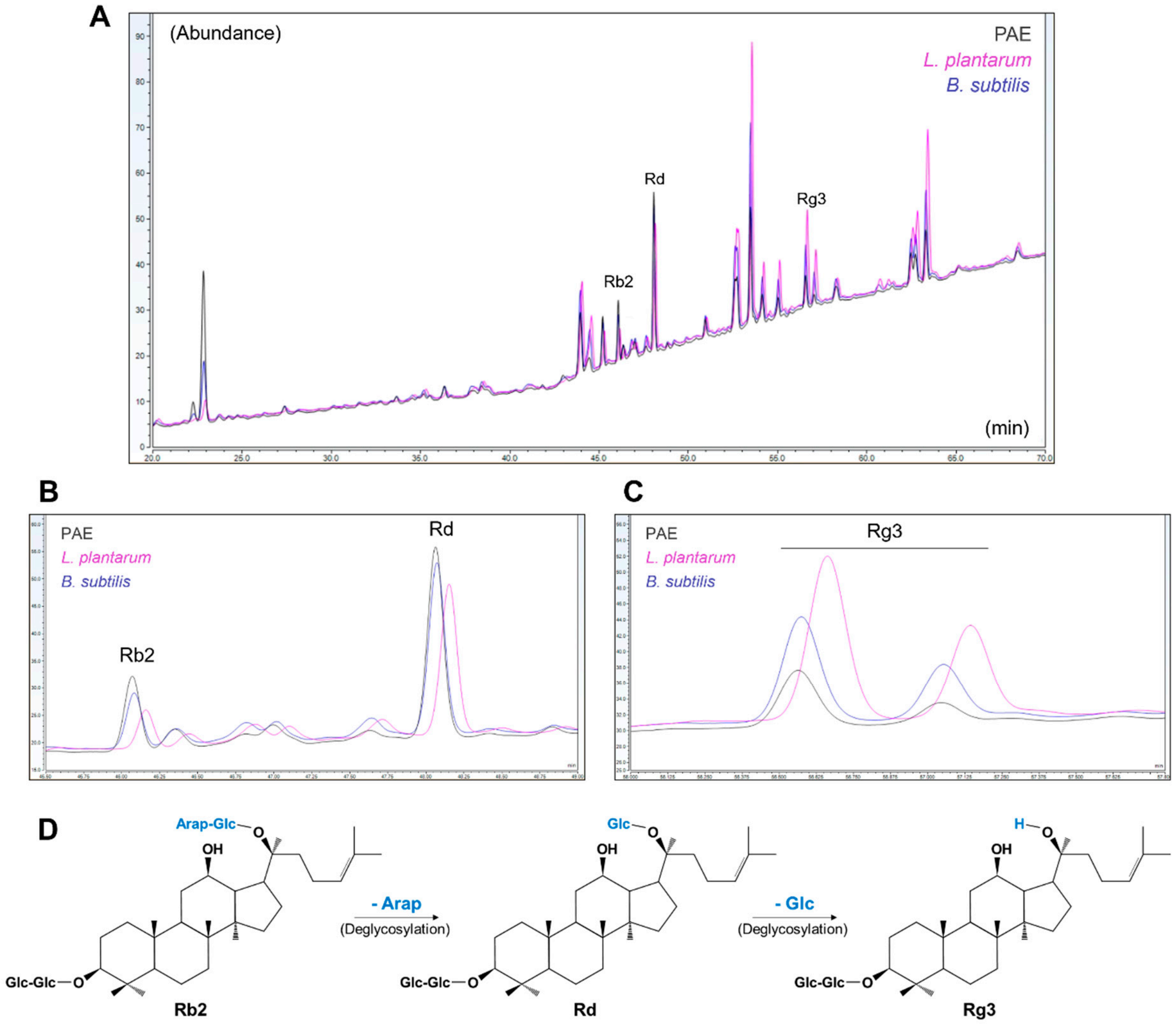

Supplementary Figure S3. Organ tissue safety assessment in ovariectomized (OVX) rats. (A) This panel shows representative images of major organ tissues, including the liver, spleen, kidney, and stomach, excised after six weeks of treatment with 150 mg/kg/day of either Rex or Rex-AG. The images provide a visual assessment of any morphological changes. (B) Depicts a comparison of the tissue mass for each organ. 17β-estradiol (E2) was utilized as a positive control. The scale bar in the images represents 1 cm. Data are present as mean ± SD (n=7). Rex, *Styphnolobium japonicum* L. fruit extract (glycoside form); Rex-AG, Rex aglycone form.

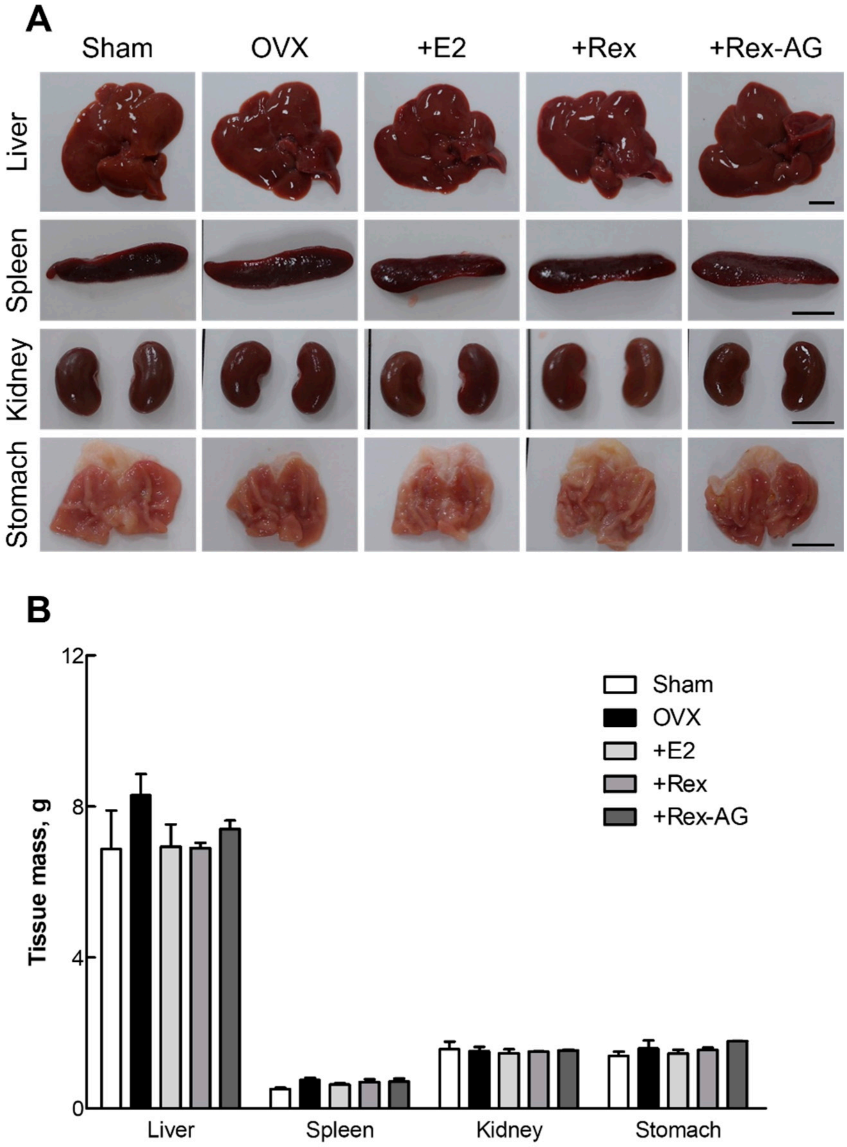

Supplement: Supplementary file 1 [file molecules-30-02218-s001.zip › molecules-3615801-supplementary.pdf]
